# A Rare Case of a Congenital Nasopharyngeal Ganglioglioma With Dyspnea in a 1-Month-Old Male Infant: A Case Report

**DOI:** 10.3389/fped.2021.690492

**Published:** 2021-06-10

**Authors:** He Zhao, Zhiwei Cao, Zhaowei Gu

**Affiliations:** Department of Otolaryngology Head and Neck Surgery, Shengjing Hospital of China Medical University, Shenyang, China

**Keywords:** nasopharyngeal, ganglioglioma, respiratory distress, infant, endoscopic resection

## Abstract

**Background:** A ganglioglioma (GG), a tumor with both neuronal and astrocytic components, rarely occurs outside the central nervous system.

**Case Summary:** We present the first reported case of a 1-month-old male with a congenital nasopharyngeal GG, nasal congestion, and dyspnea; we include the operative video. Magnetic resonance imaging was used to explore whether the tumor communicated with the intracranial space. We used an endoscopic plasma technique to ensure complete tumor resection. This afforded a good visual field, endoscopic magnification, and good hemostasis.

**Conclusions:** We report a rare case of a nasopharyngeal GG triggering nasal congestion and dyspnea in a 1-month-old male, and report our experience with the treatment of nasopharyngeal GG and similar diseases.

## Introduction

A ganglioglioma (GG) contains both astrocytic and neuronal components (1). Most GGs occur in the brain, and are rare in the pineal gland, the hypothalamus, and the optic chiasm (2). GGs develop in young subjects; the peak age at onset is between 10 and 20 years (3). To our knowledge, a GG in the nasopharynx has not been previously described. We present the rare case of a 1-month male with a congenital nasopharyngeal GG, nasal congestion, and dyspnea; we include the operative video of endoscopic plasma resection. He has been followed-up without recurrence for 3 years.

## Case Presentation

### Chief Complaints

Cough and fever for 2 day after choking on milk.

### History of Present Illness

Two days earlier, the child coughed up some milk; there was no sputum, vomiting, dyspnea, or facial cyanosis. He was brought to the Department of Neonatology. He developed fever, peaking at 37.8°C, with no shivering or convulsions. After physical cooling, the fever subsided but the body temperature rose every 4–5 h. He was reassessed in the emergency department and a chest X-ray showed that the lung was enhanced and fuzzy. His fever persisted and he was diagnosed with acute bronchopneumonia and a nasopharyngeal tumor. Two days later, he developed breathing difficulties and was unable to maintain blood oxygen saturation in response to oxygen administered *via* a nasal catheter. He was intubated, ventilated and treated with anti-inflammatory drugs.

### History of Past Illness

**Figure 1:**
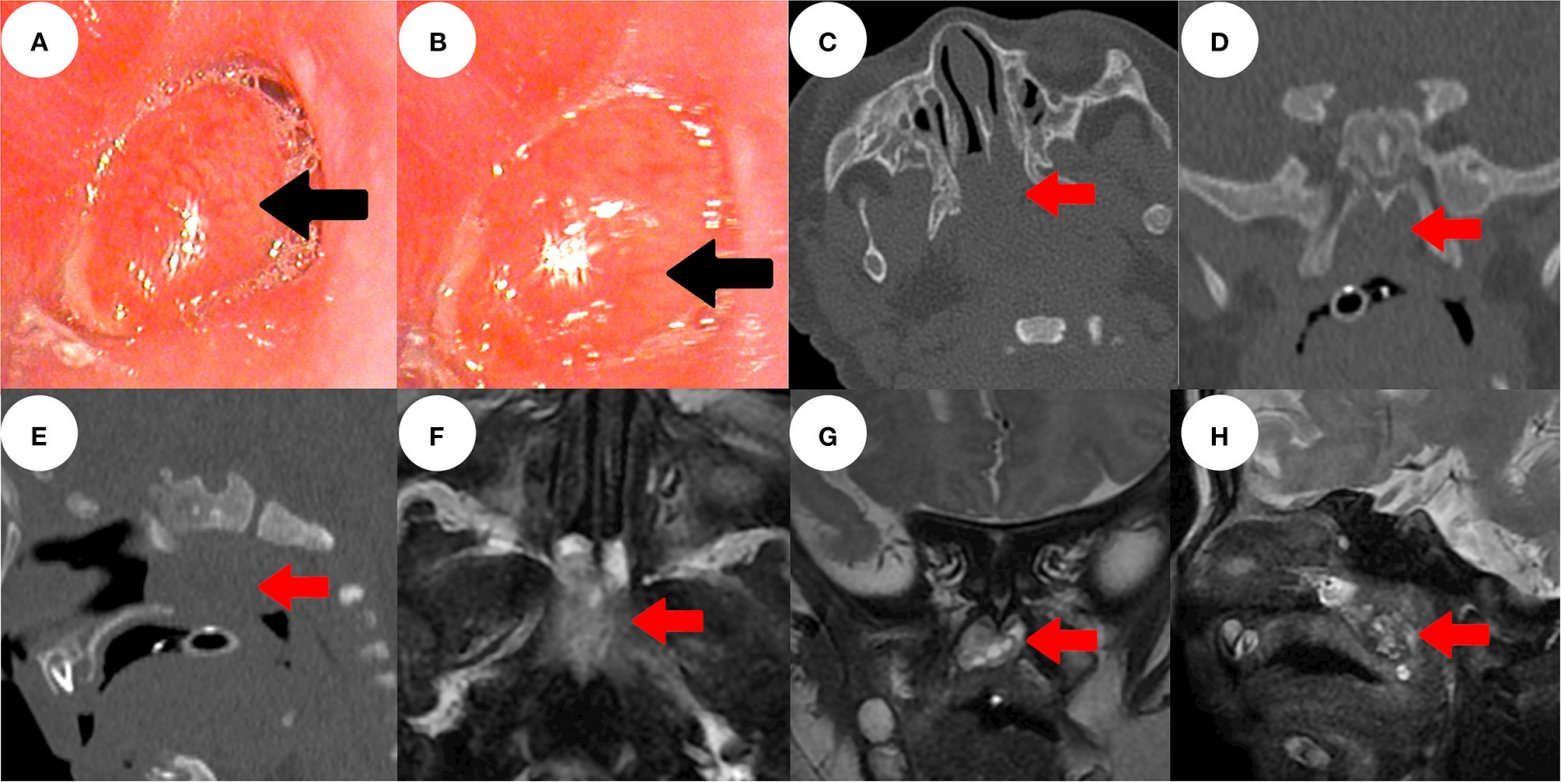
Electronic nasopharyngoscopy showed a red-pink smooth mass with an indistinct base, which almost completely blocked the posterior nostrils (black arrow) bilaterally **(A,B)**. Sinus computed tomography showed a mass with irregular soft tissue density in the nasopharynx (red arrow) **(C–E)**. Sinus magnetic resonance imaging revealed that the nasopharyngeal mass had irregular mixed signals **(F–H)**.

A 17-day-old male was delivered *via* cesarean section at full term. After birth, he breathed *via* an open mouth. There was no history of wheezing or foreign body aspiration. He underwent electronic nasopharyngoscopy because nasal obstruction was evident after birth. This revealed a red-pink, smooth nasopharyngeal mass with an unclear base that almost blocked both posterior nostrils (Figures 1A,B). As dyspnea was absent, no initial treatment was prescribed.

### Physical Examination

On the day of admission his vitals were as follows: temperature, 37.0°C; pulse, 160 times/min; respiration, 40 times/min; blood pressure, 90/61 mmHg during crying; and blood oxygen of 90% without oxygen inhalation. He was in poor general condition and was admitted with slight shortness of breath. The “nasal fan sign” and three-concave sign were absent. There was no obvious rash or bleeding and no superficial lymphadenopathy. His pupils were round (diameter = 2.5 mm) and reacted to light. There was no conjunctival congestion or cyanosis around the mouth. He breathed through his mouth and there was slight congestion of the pharyngeal isthmus. There was no herpes or ulcers, tonsillar swelling, or purulent coating. The trachea was in the midline and the was chest symmetrical, with a slight depression under the xiphoid process. Both lungs were clear on percussion, with thick breath sounds bilaterally but no wheezing. His heart sounds were strong, with an even rhythm and no obvious murmurs. His extremities were normal temperature with no hard edema or peeling of finger or toe ends. The limbs move freely. Neurological examination showed no obvious abnormalities.

### Imaging Examinations

Sinus three-dimensional computed tomography (CT) revealed a mass of irregular soft-tissue density in the nasopharynx, of dimensions ca. 2.5 × 1.2 cm, with a dark internal fluid (Figures 1C–E). Sinus contrast-enhanced magnetic resonance imaging (MRI) revealed irregular mixed signals and uneven enhancement. Nasal breathing was completely blocked; no connection was evident between the mass and the intracranial space (Figures 1F–H).

### Pathological Examinations

**Figure 2:**
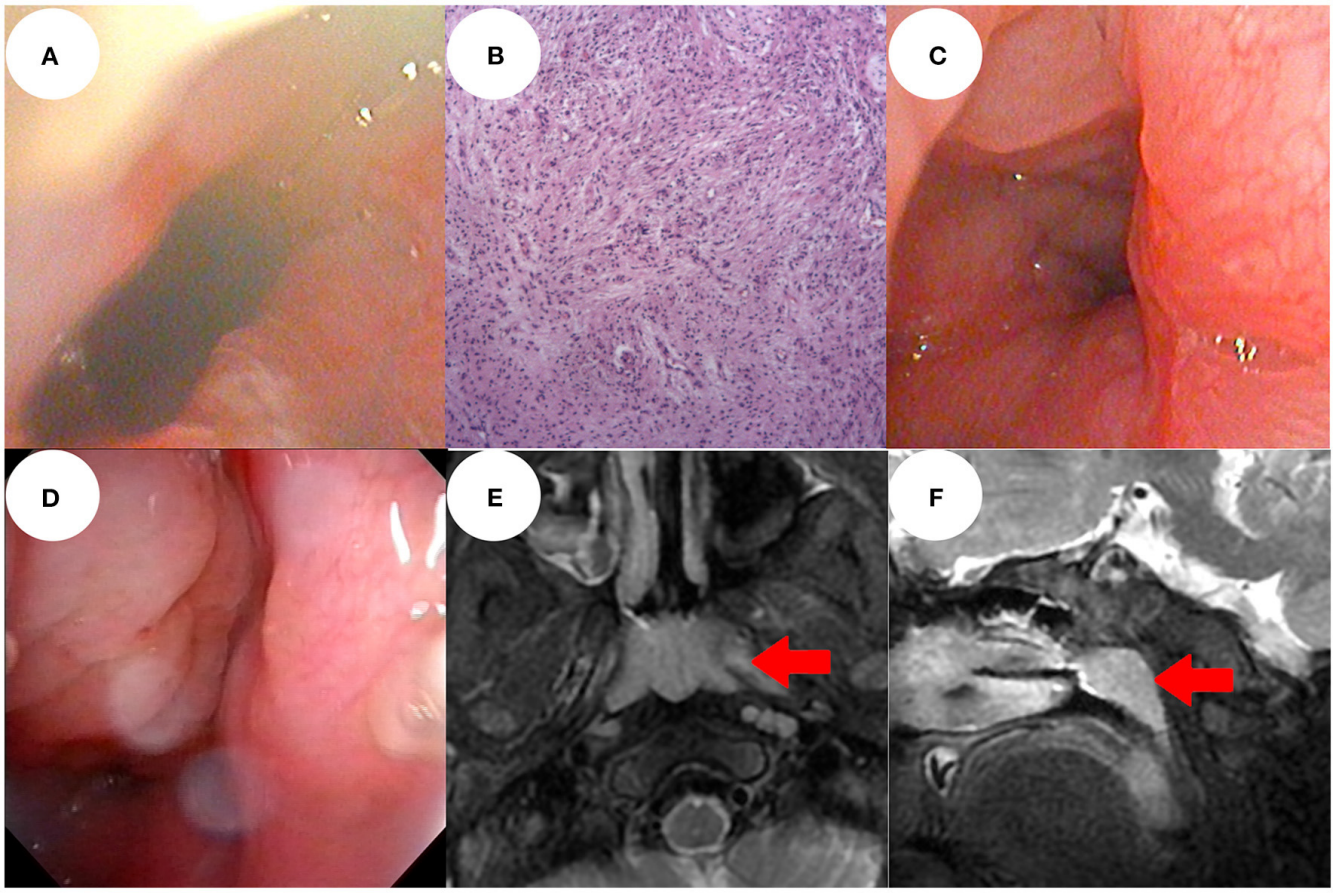
Electronic nasopharyngoscopy 1 week after surgery showed that the operation area recovered well and there was good nasopharyngeal patency **(A)**. Post-operative pathological examination was performed under a 100 × lens **(B)**. At 1 year post-operatively, electronic nasopharyngoscopy showed no recurrence of the mass, only nasopharynx adenoid hyperplasia **(C)**. At the 3-year follow-up, there was no recurrence of the mass, but obvious adenoidal hyperplasia **(D)**. Sinus magnetic resonance imaging showed no recurrence of the mass and no intracranial abnormality, only adenoidal hyperplasia (red arrow) **(E,F)**.

Pathology revealed that the mass was composed of neoplastic astrocytes and filamentous glial cells, with internal ganglion cells (Figure 2B). Immunohistochemically, the mass was S-100- and GFA-P positive, NSE- and Syn-part-positive, NeuN- and Olig-2-negative, and Ki-67-positive to the extent of about 5%. The mass was thus diagnosed as a nasopharyngeal GG.

## Final Diagnosis

Congenital nasopharyngeal GG.

### Treatment

We performed endoscopic resection with the patient under general anesthesia. A smooth nasopharyngeal mass with a clear boundary was evident on nasal endoscopy; the base lay in the left wall of the nasopharynx. We found no significant difference between the surface of the mass and that of the surrounding mucosa. We initially plasma-ablated some of the base of the mass *via* a nasal approach, but the mass was too large to be removed *via* the nasal cavity. The soft palate was pulled up with a soft catheter passed through the right nasal cavity and nasopharynx. We then plasma-cut the entire base of the mass and removed the mass from the oral cavity. Plasma ablation afforded excellent hemostasis; the wound did not require suturing and the nasal cavity did not require filling (Supplementary Video 1).

### Outcome and Follow Up

The endotracheal tube was removed on day 3 after surgery and oxygen was delivered at 1 L/min *via* a nasal catheter. The blood oxygen saturation level was normal; we recorded no dyspnea and no triple concave sign. The wound area recovered well; nasopharyngeal patency was good 1 week after surgery (Figure 2A).

One year after operation, electronic nasopharyngoscopy revealed no mass recurrence, only adenoidal hyperplasia (Figure 2C). At the 3-year follow-up, MRI and electronic nasopharyngoscopy revealed no mass recurrence and no intracranial abnormality. Endoscopic adenoidectomy was performed because the adenoidal hyperplasia interfered with sleep (Figures 2D–F).

## Discussion

A GG is a rare tumor exhibiting both neuronal and astrocytic components (4, 5). A GG may develop anywhere in the central nervous system (6); ~80% of all tumors are in the frontal and temporal lobes (7, 8). We treated a male infant a few months old. The base of the mass was located on the left lateral wall of the nasopharynx and the mass did not communicate with the intracranial space. This is rare.

The clinical manifestations of GG vary by location and size. The principal symptoms of intracranial GGs are epilepsy and intracranial hypertension (9), accompanied by headache, nausea, vomiting, endocrine symptoms, and vision problems (10). In our case, the tumor was in the nasopharynx and caused nasal obstruction and dyspnea.

GG histogenesis remains unclear; this may reflect high-level differentiation of an embryonal neuroblastoma or of residual tissue associated with a primitive neuroectodermal tumor. Others consider that the tumor reflects transformation of granular cells lying under the pia mater or the glial cells of a hamartoma (11). The histopathological hallmark of GG is the combination of neuronal and glial cell elements (12). The neuronal components are composed of well-differentiated but the atypical ganglion cells are neither primitive neuroblasts nor the small round neurons of a neuroblastoma (6). NeuN is considered to be a marker of neuronal differentiation in brain tumors, representing well-differentiated neurons to a certain extent (13), while Olig-2 is specifically expressed in oligodendrocytes (14). Nissl and immunohistochemical staining for neuronal markers (Syn, NFP, and NSE) and glial component (GFA-P or S-100) markers highlight neuronal and glial populations and greatly aid diagnosis (7, 11).

The ganglion cells are characterized by prominent nucleoli and Nissl substance and lack of a uniform orientation (7). In our case, S-100- and GFA-P positive, NSE- and Syn-part-positive, NeuN and Olig-2 were negative, show that the mass contain astrocytic and neuronal components with no mature neuron, so finally diagnosis was ganglioglioma.

The most common radiological findings are a single intraparenchymal tumor with enhanced solid and cystic components. Both calcification and restricted diffusion are rare (10). In the present case, the mass was located in the nasopharynx, was of irregular soft tissue density with a dark internal liquid, and lacked calcification. MRI is superior to CT in terms of revealing the soft tissue details and intracranial connections. In the present case, MRI yielded irregular mixed signals and revealed inhomogeneous enhancement of a nasopharyngeal mass that lacked any connection with an intracranial tissue or organ.

A GG in the nasopharynx should be differentiated from nasopharyngeal cephaloceles, Tornwaldt's cyst, adenoid mucous retention cyst, juvenile nasopharyngeal angiofibroma, rhabdomyosarcoma, and nasopharyngeal carcinoma, although these diseases are rare in the neonatal nasopharynx. The first four are the most common benign tumors of the nasopharynx in children, while rhabdomyosarcoma and nasopharyngeal carcinoma are the most common malignancies. Each tumor type has its own imaging characteristics (15–20). The imaging examination showed no communication between the tumor and brain, and there was no obvious enhancement; therefore, we ruled out nasopharyngeal cephaloceles, Tornwaldt's cyst, and juvenile nasopharyngeal angiofibroma. The final diagnosis depends on the pathology and the above diseases were excluded.

Patients with large GGs, obvious clinical symptoms, or refractory/ uncontrollable epilepsy require surgery. The optimal treatment is gross total resection (GTR); tumor location sometimes renders this impossible (10). Recurrence and malignant progression are rare (21); neither chemotherapy nor radiotherapy is required after surgery.

The surgical method should be chosen by reference to mass location and size. In our case, the mass lay in the nasopharynx. Due to the younger age, narrow nasal cavity, the operation is more difficult, once there is more bleeding, the operation will not be able to continue.

Endoscopic plasma resection exposed the surgical site clearly and was associated with good hemostasis; post-operative nasal packing was not required. Therefore, we chose the appropriate plasma to complete the operation. At the 3-year follow-up, no recurrence or intracranial abnormality was found.

To our knowledge, GG located outside the central nervous system is very rare, only 2 cases of nasal GG. Yorgancilar et al. reported a 20-year-old woman who complained of a sudden appearance of a strong yellow bloody secretion in her right nostril. A bloody and fragile mass was found in the right nasal cavity. The whole mass was removed by endoscopy assisted bipolar cauterization (2). The other case is a 14-month-old infant with an asymmetric, soft, painless tumor in the nose reaching the corner of the right eye. A pathological mass blocked the common nasal meatus. MRI showed small meningocele in the right nasal frontal area and a narrow connection with the subarachnoid space. The combined internal and external approach was used to remove the lesions (5). Our case is totally located in the left lateral wall of the nasopharynx and the mass did not communicate with the intracranial space. It is the first case with such properties.

## Conclusion

We report a rare case of a nasopharyngeal GG triggering nasal congestion and dyspnea in a 1-month-old male infant. Electronic nasopharyngoscopy and MRI/CT aid early diagnosis and define the tumor boundaries. MRI reveals any communication with the intracranial space. We performed endoscopic plasma resection; this afforded a good visual field, endoscopic magnification, and good hemostasis. Nasopharynx GG should be excluded in newborns with a smooth nasopharynx mass. We have described our experience in the treatment of GG in the newborn nasopharynx.

## Data Availability Statement

The original contributions presented in the study are included in the article/Supplementary Material, further inquiries can be directed to the corresponding author.

## Ethics Statement

The studies involving human participants were reviewed and approved by Ethics Committee of Shengjing Hospital of China Medical University. Written informed consent to participate in this study was provided by the participants' legal guardian/next of kin.

## Author Contributions

HZ and ZC co-wrote and edited the manuscript. ZG originated idea, co-wrote, and edited the manuscript. All authors contributed to the article and approved the submitted version.

## Conflict of Interest

The authors declare that the research was conducted in the absence of any commercial or financial relationships that could be construed as a potential conflict of interest.

## References

[1] Baussard B Di Rocco F Garnett MR Boddaert N Lellouch-Tubiana A Grill J. Pediatric infratentorial gangliogliomas: a retrospective series. J Neurosurg. (2007) 107(Suppl. 4):286–91. 10.3171/PED-07/10/286 17941492

[2] Yorgancilar E Yildirim M Gün R Büyükbayram H Meriç F. Ganglioglioma in the nasal cavity: a case report. Kulak Burun Bogaz Ihtis Derg. (2010) 20:267–70. 20815807

[3] Puget S Alshehri A Beccaria K Blauwblomme T Paternoster G James S. Pediatric infratentorial ganglioglioma. Childs Nerv Syst. (2015) 31:1707–16. 10.1007/s00381-015-2860-x 26351224

[4] Lundar T Due-Tønnessen BJ Fric R Egge A Krossnes B Due-Tønnessen P. Neurosurgical treatment of gangliogliomas in children and adolescents: long-term follow-up of a single-institution series of 32 patients. Acta Neurochir (Wien). (2018) 160:1207–14. 10.1007/s00701-018-3550-8 29680921 PMC5948304

[5] Niedzielska G Niedzielski A Kotowski M. Nasal ganglioglioma–difficulties in radiological imaging. Int J Pediatr Otorhinolaryngol. (2008) 72:285–7. 10.1016/j.ijporl.2007.10.019 18093665

[6] Hakim R Loeffler JS Anthony DC Black PM. Gangliogliomas in adults. Cancer. (1997) 79:127–31. 10.1002/(SICI)1097-0142(19970101)79:1<127::AID-CNCR18>3.0.CO;2-6 8988736

[7] Zentner J Wolf HK Ostertun B Hufnagel A Campos MG Solymosi L. Gangliogliomas: clinical, radiological, and histopathological findings in 51 patients. J Neurol Neurosurg Psychiatry. (1994) 57:1497–502. 10.1136/jnnp.57.12.1497 7798980 PMC1073232

[8] Varshneya K Sarmiento JM Nuño M Lagman C Mukherjee D Nuño K. A national perspective of adult gangliogliomas. J Clin Neurosci. (2016) 30:65–70. 10.1016/j.jocn.2015.12.028 27083133

[9] Antonia-Carmen L Tiberiu Augustin G Diana P Alexandru T Mihai Gheorghe L Maria S. Grading gangliogliomas: a short case series with clinico-imagistic and immunohistopathological correlations. Maedica (Bucur). (2018) 13:241–9. 10.26574/maedica.2018.13.3.241 30568746 PMC6290183

[10] Zaky W Patil SS Park M Liu D Wang WL Wani KM. Ganglioglioma in children and young adults: single institution experience and review of the literature. J Neurooncol. (2018) 139:739–47. 10.1007/s11060-018-2921-6 29882043

[11] Blümcke I Wiestler OD. Gangliogliomas: an intriguing tumor entity associated with focal epilepsies. J Neuropathol Exp Neurol. (2002) 61:575–84. 10.1093/jnen/61.7.575 12125736

[12] Wolf HK Müller MB Spänle M Zentner J Schramm J Wiestler OD. Ganglioglioma: a detailed histopathological and immunohistochemical analysis of 61 cases. Acta Neuropathol. (1994) 88:166–73. 10.1007/BF00294510 7985497

[13] Preusser M Laggner U Haberler C Heinzl H Budka H Hainfellner JA. Comparative analysis of NeuN immunoreactivity in primary brain tumours: conclusions for rational use in diagnostic histopathology. Histopathology. (2006) 48:438–44. 10.1111/j.1365-2559.2006.02359.x 16487366

[14] Preusser M Budka H Rössler K Hainfellner JA. OLIG2 is a useful immunohistochemical marker in differential diagnosis of clear cell primary CNS neoplasms. Histopathology. (2007) 50:365–70. 10.1111/j.1365-2559.2007.02614.x 17257132

[15] Rodriguez DP Orscheln ES Koch BL. Masses of the nose, nasal cavity, and nasopharynx in children. Radiographics. (2017) 37:1704–30. 10.1148/rg.2017170064 29019747

[16] Yan ZY Yang BT Wang ZC Xian JF Li M. Primary chordoma in the nasal cavity and nasopharynx: CT and MR imaging findings. AJNR. (2010) 31:246–50. 10.3174/ajnr.A1802 19797798 PMC7964161

[17] Ben Salem D Duvillard C Assous D Ballester M Krausé D Ricolfi F. Imaging of nasopharyngeal cysts and bursae. Eur Radiol. (2006) 16:2249–58. 10.1007/s00330-006-0238-x 16639497

[18] Cetinkaya EA. Thornwaldt cyst. J Craniofac Surg. (2018) 29:e560–2. 10.1097/SCS.0000000000004559 29621082

[19] Sekiya K Watanabe M Nadgir RN Buch K Flower EN Kaneda T. Nasopharyngeal cystic lesions: tornwaldt and mucous retention cysts of the nasopharynx: findings on MR imaging. J Comput Assist Tomogr. (2014) 38:9–13. 10.1097/RCT.0b013e3182a77699 24378893

[20] Lai V Khong PL. Updates on MR imaging and ^18^F-FDG PET/CT imaging in nasopharyngeal carcinoma. Oral Oncol. (2014) 50:539–48. 10.1016/j.oraloncology.2013.05.005 23769923

[21] Bowles AP Jr Pantazis CG Allen MB Jr Martinez J Allsbrook WC Jr. Ganglioglioma, a malignant tumor? Correlation with flow deoxyribonucleic acid cytometric analysis. Neurosurgery. (1988) 23:376–81. 10.1227/00006123-198809000-00019 3226518

